# Cost-Effectiveness of Temporary Financial Assistance for Veterans Experiencing Housing Instability

**DOI:** 10.1001/jamanetworkopen.2024.43396

**Published:** 2024-11-05

**Authors:** Richard E. Nelson, Alec Chapman, Thomas Byrne, Nathorn Chaiyakunapruk, Ying Suo, Atim Effiong, Warren Pettey, Lillian Gelberg, Stefan G. Kertesz, Jack Tsai, Ann Elizabeth Montgomery

**Affiliations:** 1 Informatics, Decision-Enhancement and Analytic Sciences Center, Veterans Affairs Salt Lake City Health Care System, Salt Lake City, Utah; 2 Department of Internal Medicine, University of Utah School of Medicine, Salt Lake City; 3 National Center on Homelessness Among Veterans, Washington, DC; 4 School of Social Work, Boston University, Boston, Massachusetts; 5 Center for Healthcare Organization and Implementation Research, Bedford Veterans Affairs Medical Center, Bedford, Massachusetts; 6 Department of Pharmacotherapy, University of Utah College of Pharmacy, Salt Lake City; 7 Department of Family Medicine, David Geffen School of Medicine at UCLA, Los Angeles, California; 8 Office of Healthcare Transformation and Innovation, Veterans Affairs Greater Los Angeles Healthcare System, Los Angeles, California; 9 Department of Health Policy & Management, Fielding School of Public Health, University of California at Los Angeles, Los Angeles; 10 Birmingham Veterans Affairs Health Care System, Birmingham, Alabama; 11 Department of Preventive Medicine, Heersink UAB School of Medicine, Birmingham, Alabama; 12 School of Public Health, University of Texas Health Science Center at Houston, Houston; 13 School of Public Health, University of Alabama at Birmingham, Birmingham

## Abstract

**Importance:**

The US Department of Veterans Affairs (VA) partners with community organizations (grantees) across the US to provide temporary financial assistance (TFA) to vulnerable veterans through the Supportive Services for Veteran Families (SSVF) program. The goal of TFA for housing-related expenses is to prevent homelessness or to quickly house those who have become homeless.

**Objective:**

To assess the cost-effectiveness of the SSVF program with TFA vs without TFA as an intervention for veterans who are experiencing housing insecurity.

**Design, Setting, and Participants:**

This study used a Markov simulation model to compare cost and housing outcomes in a hypothetical cohort of veterans enrolled in the SSVF program. Enrollees who are homeless receive rapid rehousing services, while those who are at risk of becoming homeless receive homelessness prevention services.

**Exposure:**

The SSVF program with TFA for veterans who are experiencing housing insecurity.

**Main Outcomes and Measures:**

The effectiveness measure was the incremental cost-effectiveness ratio (ICER) with quality-adjusted life-years (QALYs). The model was parameterized using a combination of inputs taken from published literature and internal VA data. The model had a 2-year time horizon and a 1-day cycle length. In addition, probabilistic sensitivity analyses were conducted using 10 000 Monte Carlo simulations.

**Results:**

The base case analyses found that the SSVF program with TFA was more costly ($35 814 vs $32 562) and yielded more QALYs (1.541 vs 1.398) than the SSVF program without TFA. The resulting ICER was $22 676 per QALY, indicating that TFA is the preferred strategy at a willingness-to-pay threshold of $150 000 per QALY. This ICER was $19 114 per QALY for veterans in the rapid rehousing component of the SSVF program and $29 751 per QALY for those in the homelessness prevention component of the SSVF program. At a willingness-to-pay threshold of $150 000 per QALY, probabilistic sensitivity analyses showed that TFA was cost-effective in 8972 of the 10 000 Monte Carlo simulations (89.7%) for rapid rehousing and in 8796 of the 10 000 Monte Carlo simulations (88.0%) for homelessness prevention only.

**Conclusions and Relevance:**

This economic evaluation suggests that TFA is a cost-effective approach (ie, yields improved health benefits at a reasonable cost) for addressing housing insecurity for veterans enrolling in the SSVF program. Future research could examine the cost effectiveness of large, nationwide housing interventions such as this one among subpopulations of veterans such as those with certain comorbidities including severe mental illness or substance use disorders, those with chronic diseases, or those experiencing long-term housing instability vs acute loss of housing.

## Key Points

**Question:**

Is it cost-effective from the perspective of the Department of Veterans Affairs to provide temporary financial assistance (TFA) for veterans experiencing housing instability who are enrolled in the Supportive Services for Veteran Families (SSVF) program?

**Findings:**

This cost-effectiveness analysis using a Markov simulation model found that the SSVF program with TFA was more costly and yielded more quality-adjusted life-years (QALYs) than the SSVF program without TFA, resulting in an incremental cost-effectiveness ratio of $22 676 per QALY.

**Meaning:**

This study suggests that TFA is a cost-effective strategy for providing assistance to veterans at a willingness-to-pay threshold of $150 000 per QALY.

## Teaser

This economic evaluation assesses the cost-effectiveness of the US Supportive Services for Veteran Families program with temporary financial assistance for housing-related expenses vs without temporary financial assistance for veterans who are experiencing housing insecurity.

## Introduction

Over the past several decades, there has been growing recognition of the important influence that social determinants of health (SDOHs) can play in patient health outcomes.^1^ This has led to calls from high-profile governing bodies for health care professionals and payers to address these SDOHs to improve health equity and control health care costs.^2,3,4^ Many health systems have heeded these calls; a recent study estimated that between 2017 and 2019, 78 unique programs from 57 different health systems in the US invested $2.5 billion in programs designed to improve SDOHs.^5^ Housing programs accounted for $1.6 billion, or almost two-thirds of this funding.

In fiscal year 2023, the US federal government spent $8.7 billion on targeted housing assistance programs for individuals experiencing homelessness.^6^ Many of these programs use the Housing First approach, in which the primary goal is to provide an individual experiencing homelessness with permanent housing. Housing assistance programs supported by the federal government include both long-term (ie, permanent supportive housing) and short-term (ie, rapid rehousing) programs.

The Department of Veterans Affairs (VA) administers programs of both types, including the US Department of Housing and Urban Development-VA Supportive Housing (HUD-VASH) program, which provides permanent supportive housing, and the Supportive Services for Veteran Families (SSVF) program, which includes rapid rehousing. An additional component of the SSVF program is homelessness prevention, which is intended for veterans who are not homeless but at risk of becoming so in the immediate future. The SSVF program is a partnership between the VA and nonprofit organizations nationwide providing case management, assistance accessing both VA and non-VA health benefits, and health care navigation services. One service offered through the SSVF program is temporary financial assistance (TFA), which is a short-term monetary benefit that can be used to pay rent, utility payments, security deposits, and other housing-related expenses that the veteran may have. Previous studies have found that TFA is associated with higher rates of stable housing,^7^ lower health care costs,^8^ and lower rates of mortality and suicidal ideation.^9^

Social services organizations—whether at the national level like the VA or at the local, state, or municipal level—have many competing priorities and limited financial resources with which to help individuals facing housing instability. Rigorous economic evaluations of social services interventions are vital to inform policy makers as to the best use of these limited funds to be able to have the most effect. Although more common in health care settings, cost-effectiveness analyses can play a key role in social services evaluations as well, as they can allow for quantifying the tradeoff between benefits that recipients would receive and the resources required to produce those benefits.

Only a handful of cost-effectiveness analyses of homelessness interventions exist in the published literature, to our knowledge; those that do exist report results from clinical trials, one focused on the HUD-VASH program in the VA population^10^ and 2 others focused on Housing First interventions in Canada^11^ and France.^12^ Although randomized clinical trials are considered the criterion standard for study design due to their internal validity and lack of bias in estimates due to unmeasured confounding, studies using observational data can have major advantages such as greater external validity and larger sample sizes. In this article, we describe an economic evaluation of TFA through the SSVF program to compare the costs associated with the program and the benefits received by the veterans using a simulation model parameterized with veteran data from encounters with the SSVF program.

## Methods

### Overview and Model Structure

**Figure 1.**
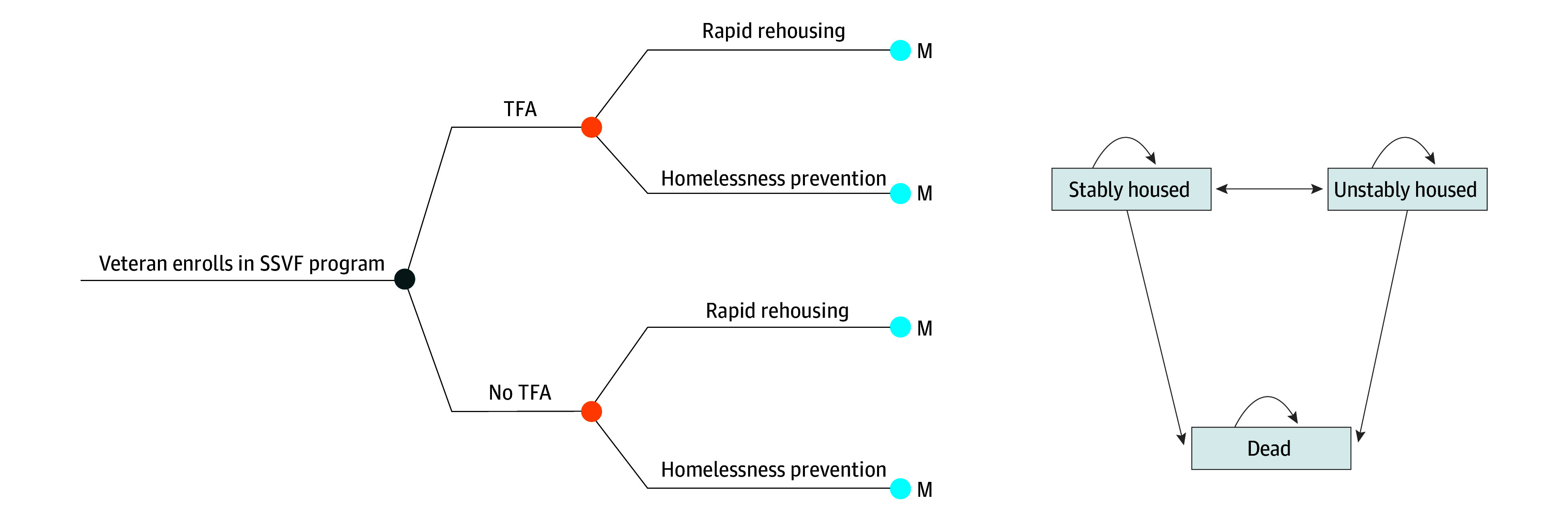
Markov Simulation Model SSVF indicates Supportive Services for Veteran Families; and TFA, temporary financial assistance. The M and light blue circle indicate a Markov node.

For this study, we constructed a Markov simulation model in TreeAge Pro Healthcare 2022 (TreeAge LLC) (Figure 1), to compare costs and health benefits of receiving TFA compared with not receiving TFA during an SSVF program episode. Simulated veterans entered this model by enrolling in the SSVF program. Depending on whether they were homeless at the time of enrollment or at imminent risk of becoming homeless, they enrolled in the rapid rehousing component or homelessness prevention component of the SSVF program, respectively. At that point in the model, the veteran entered a Markov node in which they transitioned between stable housing, unstable housing, and death health states in daily cycles. Effectiveness was measured by (1) days of stable housing and (2) quality-adjusted life-years (QALYs), a commonly used approach in economic evaluations. Quality-adjusted life-years are constructed by applying utility weights that vary between 0 (death) and 1 (perfect health) to time spent in particular health states. We ran our model with a 2-year time horizon and applied a discount rate of 3% on outcomes occurring in the second year. We analyzed our model from the VA perspective with costs valued in 2022 US dollars. Costs were adjusted to 2022 US dollars using the Personal Health Care price index.^13^ This study was approved by the institutional review board at the University of Utah. The reporting of this study conforms to the Consolidated Health Economic Evaluation Reporting Standards (CHEERS) statement for economic analyses (eAppendix in Supplement 1).

### Input Parameters

Input parameters for our model are listed in Table 1.^7,8,9,14,15^ After a veteran exited from the SSVF program, the probability of stable housing in our model was based on a previously published analysis that estimated the positive association of TFA with stable housing.^7^ With the initial designation of stable or unstable housing as a starting point, we estimated transition probabilities between stable housing, unstable housing, and death health states using the multistate model approach in R, version 4.4.1 (R Project for Statistical Computing).^16^ The data for this multistate model were extracted from the VA Corporate Data Warehouse. Death dates were obtained from the VA Corporate Data Warehouse vital records. Housing status was extracted from clinical text using a previously developed and validated natural language processing (NLP) system that characterized a veteran’s housing stability from free text notes recorded in the VA electronic health record.^17^ In particular, we used this NLP system to identify all mentions of stable and unstable housing in free text in the electronic health record over the 2 years after exit from the SSVF program. Veterans are seen intermittently by health care professionals in the VA. To account for long gaps between measurements, we used a continuous time multistate modeling framework. In continuous time multistate models, transitions are assumed to occur at any point in a range of time, which may or may not be exactly observed in the data. Transition probabilities (eFigure 1 and eFigure 2 in Supplement 1) were assumed to be constant within the following time windows: 0 to 90 days, 91 to 180 days, 181 to 365 days, and 366 to 730 days. See eAppendix, eFigure 3, and eFigure 4 in Supplement 1 for more details on the multistate model, including validation.

**Table 1.** Input Parameters by Supportive Services for Veteran Families Program Component

Input	Homelessness prevention			Rapid rehousing			Source
	Value	Low	High	Value	Low	High	
Probabilities							
Stable housing, no TFA	0.821	NA	NA	0.492	NA	NA	Nelson et al,^7^ 2021
Risk difference for stable housing, TFA vs no TFA	0.112	0.090	0.127	0.301	0.288	0.315	Nelson et al,^7^ 2021
Mortality per month, unstable housing	0.0032	0.0016	0.0049	0.0032	0.0016	0.0049	Schinka et al,^14^ 2018
Mortality per month, stable housing	0.0015	0.0010	0.0021	0.0015	0.0010	0.0021	Schinka et al,^14^ 2018
Relative risk of mortality, TFA vs no TFA	0.838	0.723	0.971	0.838	0.723	0.971	Nelson et al,^9^ 2024
Costs							
Quarterly health care cost, no TFA, $	3636	NA	NA	4551	NA	NA	Nelson et al,^8^ 2021
Change in quarterly health care cost, TFA vs no TFA, $	–227	–444	–11	–475	–659	–293	Nelson et al,^8^ 2021
Cost of TFA, $	5931	NA	NA	6268	NA	NA	Nelson et al,^8^ 2021
Utility weights							
Unstable housing	0.434	0.269	0.473	0.434	0.269	0.473	Rajan et al,^15^ 2021
Stable housing	1	0.800	1	1	0.800	1	Assumption

Abbreviations: NA, not applicable; TFA, temporary financial assistance.

We also explored an alternative specification for transitions from stable and unstable housing to mortality from a published study that used data from a non-VA population.^14^ In this specification, the risk of mortality for TFA recipients relative to non-TFA recipients was based on a recent study of the SSVF program.^9^

The utility weight for unstable housing was assumed to be 0.434, as derived from a recently published study that used a standard gamble survey administered to 6607 middle- and low-income adults in the US.^15^ In addition, we obtained several input parameters from previous studies that focused specifically on the associations of TFA with veteran outcomes. For example, we obtained probabilities of stable housing at the time of exit from the SSVF program for those not receiving TFA (0.821 for those in the homelessness prevention component and 0.492 for those in the rapid rehousing component) and the risk differences for stable housing for those receiving TFA (0.112 [95% CI, 0.090-0.127] for those in the homelessness prevention component and 0.301 [95% CI, 0.288-0.315] for those in the rapid rehousing component).^7^

We included the mean dollar amount of TFA allocated to SSVF program enrollees as cost of the intervention. This was estimated to be $5931 for homelessness prevention and $6268 for the rapid rehousing component.^8^ We also included the direct medical costs to the VA for health care utilization that is associated with receiving TFA after SSVF program enrollment. These estimates were obtained from an observational study of SSVF program enrollees that used a difference-in-differences approach.^8^ The mean baseline quarterly health care costs in this population were $3636 for homelessness prevention and $4551 for the rapid rehousing component, while TFA is associated with a $227 (95% CI, $11-$444) decrease in quarterly health care costs for homelessness prevention and $475 (95% CI, $293-$659) decrease in quarterly health care costs for rapid rehousing.^8^ We did not include incarceration in our model, as a systematic review indicated that Housing First interventions have little association with criminal justice involvement.^18^

### Statistical Analysis

In our primary analyses, we ran our simulation model to obtain estimates of the incremental cost and incremental effectiveness of receiving TFA compared with receiving no TFA for all SSVF enrollees. These estimates were then combined to create an incremental cost-effectiveness ratio (ICER), which quantifies the tradeoff between dollars and effectiveness measures (ie, QALYs and days of stable housing). More precisely, in this specific analysis, the ICER measures the extra cost incurred by the VA when a veteran enrolling in the SSVF program receives TFA to gain 1 additional QALY or day of stable housing compared with not receiving TFA. We also performed these calculations separately for enrollees in the rapid rehousing and homelessness prevention components of the SSVF program separately. A willingness-to-pay threshold of $150 000 per QALY was used to assess cost effectiveness.^19,20^

We explored the sensitivity of our model results by examining ranges of values for various input parameters. In 1-way sensitivity analyses, we varied the value of the utility weight for unstable housing from 0.45 to 0.95. Finally, we conducted probabilistic sensitivity analyses using 10 000 Monte Carlo simulations in which values for each parameter were randomly drawn from a distribution. Beta distributions were used for probabilities and utility weights and gamma distributions were used for costs.

## Results

In our base case analyses, the TFA strategy was associated with greater costs ($35 814 vs $32 562), greater QALYs (1.541 vs 1.398), and greater days in stable housing (447.0 vs 356.4) compared with the no TFA strategy for all SSVF program enrollees (Table 2). This strategy resulted in ICERs of $22 676 per QALY and $35.91 per day of stable housing. For the subgroup of veterans enrolling in the rapid rehousing component of the SSVF program, the incremental cost was slightly lower than those enrolling in the homelessness prevention component ($2733 vs $4291). The incremental effectiveness was similar between the 2 groups. The resulting ICERs were $19 114 per QALY and $30.21 per day of stable housing for the rapid rehousing component and $29 751 per QALY and $47.31 per day of stable housing for the homelessness prevention component. ICERs were similar using the alternative specification for transitions to death.

**Table 2.** Cost-Effectiveness Analysis Results

Strategy	Cost,$	Effectiveness		Incremental cost, $	Incremental effectiveness		Incremental cost-effectiveness ratio	
		No. of days of stable housing	QALYs		No. of days of stable housing	QALYs	$/Day of stable housing	$/QALY
Overall								
No TFA	32 562	356.4	1.398	NA	NA	NA	NA	NA
TFA	35 814	447.0	1.541	3252	90.6	0.143	35.91	22 676
Rapid rehousing only								
No TFA	34 823	352.5	1.390	NA	NA	NA	NA	NA
TFA	37 556	442.9	1.533	2733	90.5	0.143	30.21	19 114
Homelessness prevention only								
No TFA	28 033	364.4	1.415	NA	NA	NA	NA	NA
TFA	32 325	455.1	1.559	4291	90.7	0.144	47.31	29 751

Abbreviations: NA, not applicable; QALY, quality-adjusted life-year; TFA, temporary financial assistance.

**Figure 2.**
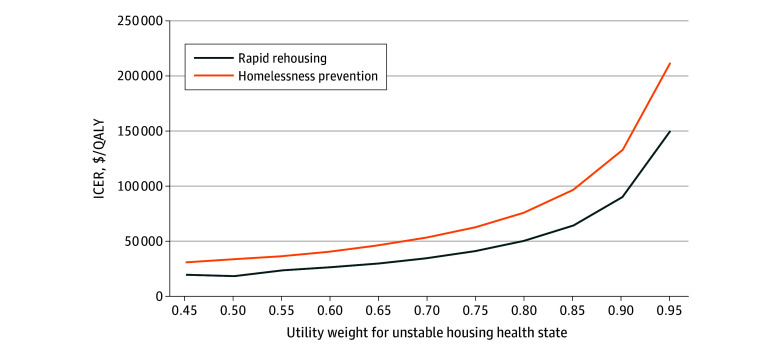
One-Way Sensitivity Analyses of Utility Weight for Unstable Housing Association between incremental cost-effectiveness ratio (ICER) and utility weight for unstable housing. QALY indicates quality-adjusted life-year.

The results from our 1-way sensitivity analyses of the unstable housing utility weight are shown in Figure 2, which shows ICERs for different utility weight values for rapid rehousing and homelessness prevention SSVF program enrollees. The ICER was below $150 000 per QALY for each utility weight value up to 0.95 for rapid rehousing and up to 0.90 for homelessness prevention.

**Figure 3.**
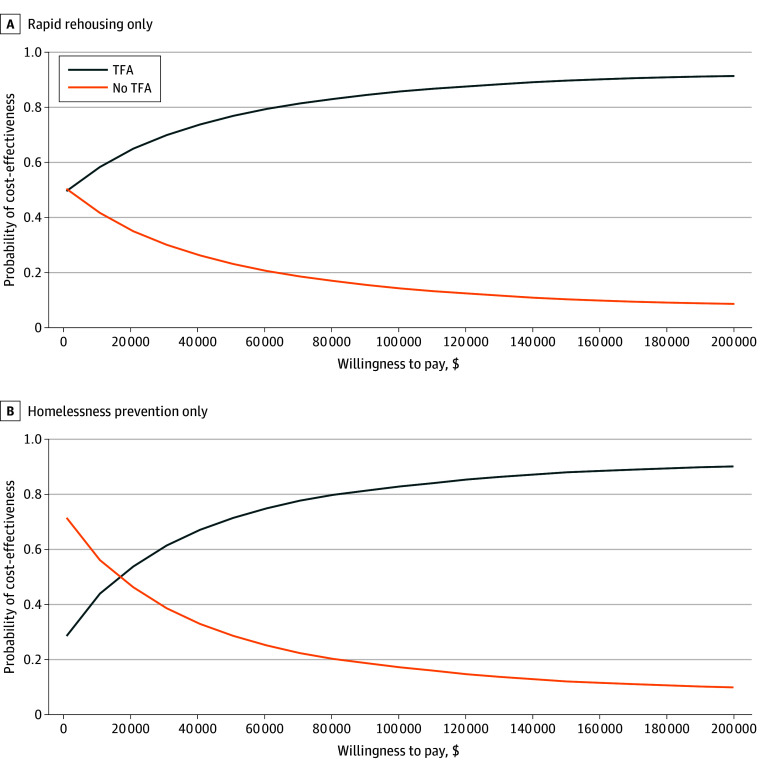
Cost-Effectiveness Acceptability Curves A, Rapid rehousing component only. B, Homelessness prevention component only. Association between willingness-to-pay threshold and proportion of Monte Carlo simulations in which the strategies were deemed cost-effective. TFA indicates temporary financial assistance.

Figure 3 shows the results of our probabilistic sensitivity analyses as cost-effectiveness acceptability curves that depict the percentage of Monte Carlo simulations in which each strategy was cost effective at various willingness-to-pay thresholds. At our willingness-to-pay threshold of $150 000 per QALY, TFA was cost effective in 8972 of the iterations (89.7%) for rapid rehousing and 8796 of the iterations (88.0%) for homelessness prevention only.

## Discussion

In this economic evaluation of TFA within the VA SSVF program, we compared the costs, QALYs, and days of stable housing for individuals facing housing instability who did and did not receive TFA. We found that TFA was a cost-effective strategy for providing housing support to veterans experiencing homelessness or who were at risk of becoming homeless. Relative to the no TFA group, the incremental cost of providing TFA was lower and the incremental effectiveness associated with TFA was larger for SSVF program enrollees in the rapid rehousing component compared with those in the homelessness prevention component. Although this yielded a lower ICER for rapid rehousing compared with homelessness prevention, in both cases, the ICER was well below the willingness-to-pay threshold of $150 000 per QALY. In other words, compared with no TFA, the increase in health benefits of TFA—as measured by QALYs—outweighed the increase in cost of TFA. These findings were robust and consistent across both deterministic and probabilistic sensitivity analyses.

Although our analysis focused on 1 specific VA housing intervention, albeit one that has a $700 million annual budget and is national in scope, our findings are broadly applicable to other housing programs. For instance, renter households were disproportionately affected by unemployment after the COVID-19 pandemic. The US government’s Coronavirus Aid, Relief, and Economic Security Act of March 2020 provided $3.9 billion for emergency rental assistance through COVID-19 relief funds and community development block grants.^21^ Most of this initial allotment provided financial assistance, such as rent and utility payments, for individuals who had low incomes, experienced financial difficulties as a result of the pandemic, and experienced housing instability.^22^ Several months later, the US government’s Emergency Rental Assistance program was created in late December 2020, allocating $25 billion for a broad range of housing-related expenses. An additional $21.55 billion was added to this amount in March 2021 through the American Rescue Plan Act. These funds were used to make more than 10 million payments, primarily for low-income renters, with the goal of helping these individuals maintain stable housing by preventing evictions,^23^ similar to the homelessness prevention component of the SSVF program. A recent study has found that emergency rental assistance is associated with increases in housing stability as well as mental health and financial well-being.^24^ Our findings indicate that emergency rental assistance funds may also be a cost-effective intervention to stabilize housing, although this possibility would need to be explored in a research study of its own.

Our results are in line with those from the few cost-effectiveness analyses of housing interventions that exist in the published literature, all of which report results from randomized clinical trials. For instance, a 2003 study by Rosenheck and colleagues^10^ reports a cost-effectiveness analysis of supported housing through HUD-VASH, alongside a clinical trial that compared HUD-VASH vouchers and case management, case management alone, and usual care. They found that patients in the HUD-VASH arm had more housed days as well as greater costs from a societal perspective than the other 2 arms. Latimer et al^11^ conducted a cost-effectiveness analysis alongside the At Home/Chez Soi clinical trial that examined a Housing First intervention with intensive case management for homeless individuals with mental illness in Canada. This intervention yielded an increase in days housed and a positive net cost relative to treatment as usual. Finally, Lemoine et al^12^ conducted a cost-effectiveness analysis of a similar Housing First intervention in France using a Markov simulation model. The authors used a randomized trial of this intervention to parameterize the model and, similar to the previous studies, found that Housing First yielded a greater number of days housed at a positive incremental cost.

### Limitations and Strengths

Our study has several limitations. First, because the intervention we were evaluating is specific to the VA health care system and is, therefore, only available to US veterans facing housing instability, our results may not be directly generalizable to a non-VA audience. However, as stated above, our analysis could provide a template for economic evaluations of other housing interventions, thus strengthening the evidence base in this area. Second, we focused on the health-related quality of life benefits of stable housing in our analysis, but it is important to remember that better housing situations can improve many other aspects of individuals’ lives, so our results represent an underestimate of the benefits that TFA may produce.^25^ Third, the housing status variable used in our multistate model was observed at irregular times and extracted using NLP, which is an imperfect measure of the true housing status.

Despite these limitations, our study also had many important strengths. For instance, it is one of just a few economic evaluations of housing interventions for homeless individuals and the first, to our knowledge, that uses real-world data from observational studies as input parameters. In addition, the SSVF program that we evaluated, and the TFA component of it specifically, can be seen as a model for other housing interventions that are either being considered or have already been implemented. For this reason, our findings of cost effectiveness for TFA bode well for the tradeoffs associated with these other programs. Finally, the intervention studied was implemented on a large geographic scale.

## Conclusions

In this economic evaluation of TFA in the SSVF program, we found that providing short-term financial assistance to veterans facing housing instability can be associated with improved rates of stable housing and lower health care costs. We found that these lower health care costs were not enough to entirely offset the cost of the financial assistance itself but that the health-related quality of life associated with increased stable housing outweighed the net increase in cost. We also found lower ICERs for the rapid rehousing component of the SSVF program than the homelessness prevention component, although both were below the willingness-to-pay threshold of $150 000 per QALY. Future research could examine the cost effectiveness of a large, nationwide housing interventions, such as this one among subpopulations of veterans such as those with certain comorbidities including severe mental illness or substance use disorders or those experiencing long-term housing instability vs acute loss of housing. In addition, due to a high degree of variability in local housing availability and affordability, patient heterogeneity, and grantee engagement, TFA may be cost effective in some settings and not in others. For this reason, conducting these evaluations at more granular units—including different regions in the US, urban vs rural areas, and even at the individual grantee level—would provide VA policymakers with valuable information as to how to target resources to improve efficiency and performance.

## Notes

Supplement 1. **eFigure 1.** Transition Probabilities Generated From Multistate Models for Rapid Rehousing SSVF Enrollees **eFigure 2.** Transition Probabilities Generated From Multistate Models for Homelessness Prevention SSVF Enrollees **eAppendix.** Details of Modeling Approach **eFigure 3.** Multi-State Model Between Stable Housing, Unstable Housing, and Death **eFigure 4.** Comparison of State Probabilities From Markov Model With Weighted and Unweighted Marginal Longitudinal Models
eReferences.

Supplement 2.
Data Sharing Statement
