# Supplementary material for: Cost-Effectiveness of Temporary Financial Assistance for Veterans Experiencing Housing Instability
Source: JAMA Netw Open. 2024 Nov 5;7(11):e2443396. doi: 10.1001/jamanetworkopen.2024.43396 (PMC11539017; doi:10.1001/jamanetworkopen.2024.43396)
Supplement: Supplement 1. — eFigure 1. Transition Probabilities Generated From Multistate Models for Rapid Rehousing SSVF Enrollees eFigure 2. Transition Probabilities Generated From Multistate Models for Homelessness Prevention SSVF Enrollees eAppendix. Details of Modeling Approach eFigure 3. Multi-State Model Between Stable Housing, Unstable Housing, and Death eFigure 4. Comparison of State Probabilities From Markov Model With Weighted and Unweighted Marginal Longitudinal Models eReferences. [file jamanetwopen-e2443396-s001.pdf]

Supplementary Online Content

Nelson RE, Chapman A, Byrne T, et al. Cost-effectiveness of temporary financial assistance for veterans experiencing housing instability. *JAMA Netw Open*. 2024;7(11):e2443396. doi:10.1001/jamanetworkopen.2024.43396

eFigure 1. Transition Probabilities Generated from Multistate Models for Rapid Rehousing SSVF Enrollees
eFigure 2. Transition Probabilities Generated from Multistate Models for Homelessness Prevention SSVF Enrollees
eAppendix. Details of Modeling Approach
eFigure 3. Multi-State Model Between Stable Housing, Unstable Housing, and Death
eFigure 4. Comparison of State Probabilities from Markov Model With Weighted and Unweighted Marginal Longitudinal Models
eReferences.

This supplementary material has been provided by the authors to give readers additional information about their work.

eFigure 1. Transition Probabilities Generated from Multistate Models for Rapid Rehousing SSVF Enrollees

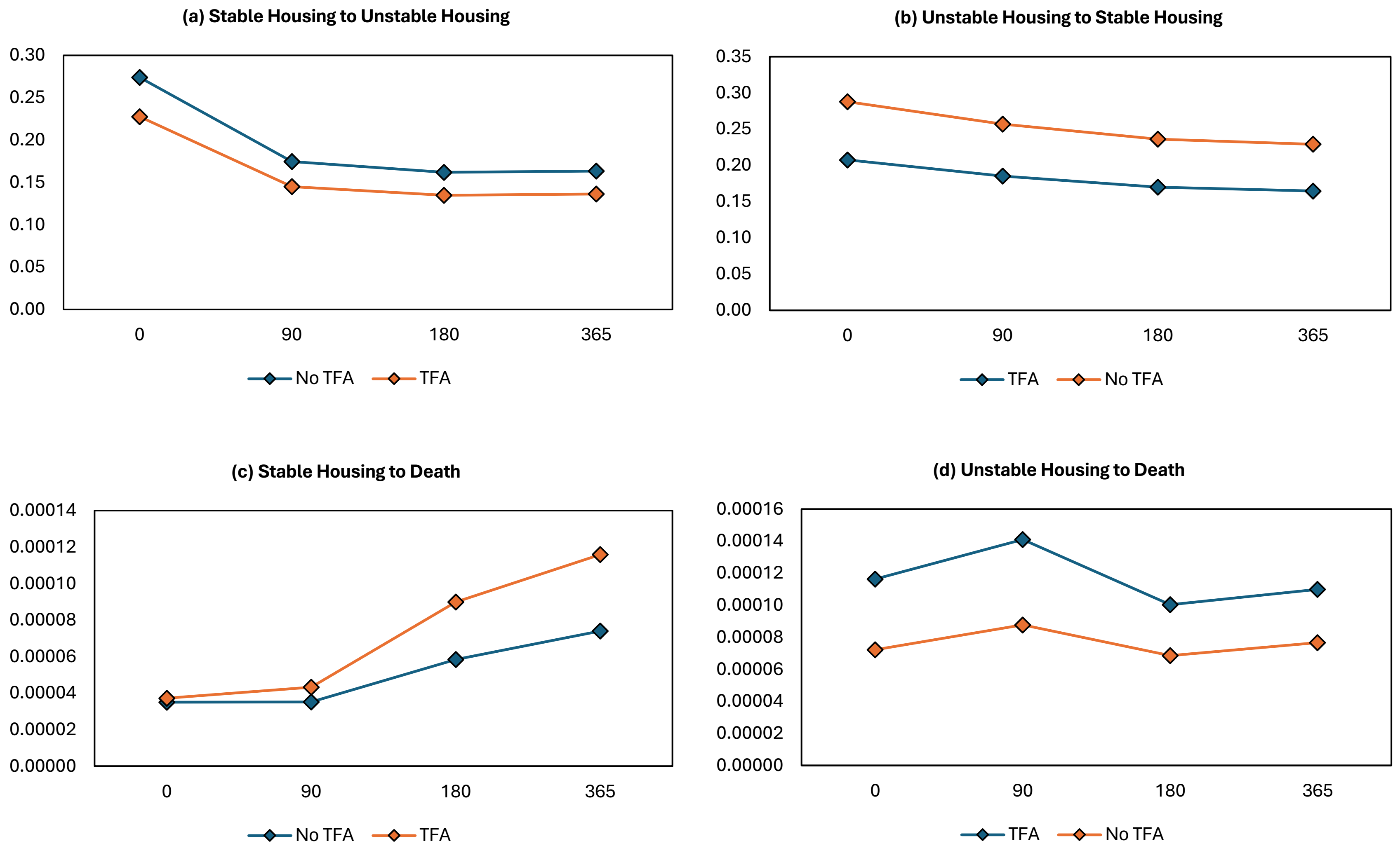

© 2024 Nelson RE et al. *JAMA Network Open.*

eFigure 2. Transition Probabilities Generated from Multistate Models for Homelessness Prevention SSVF Enrollees

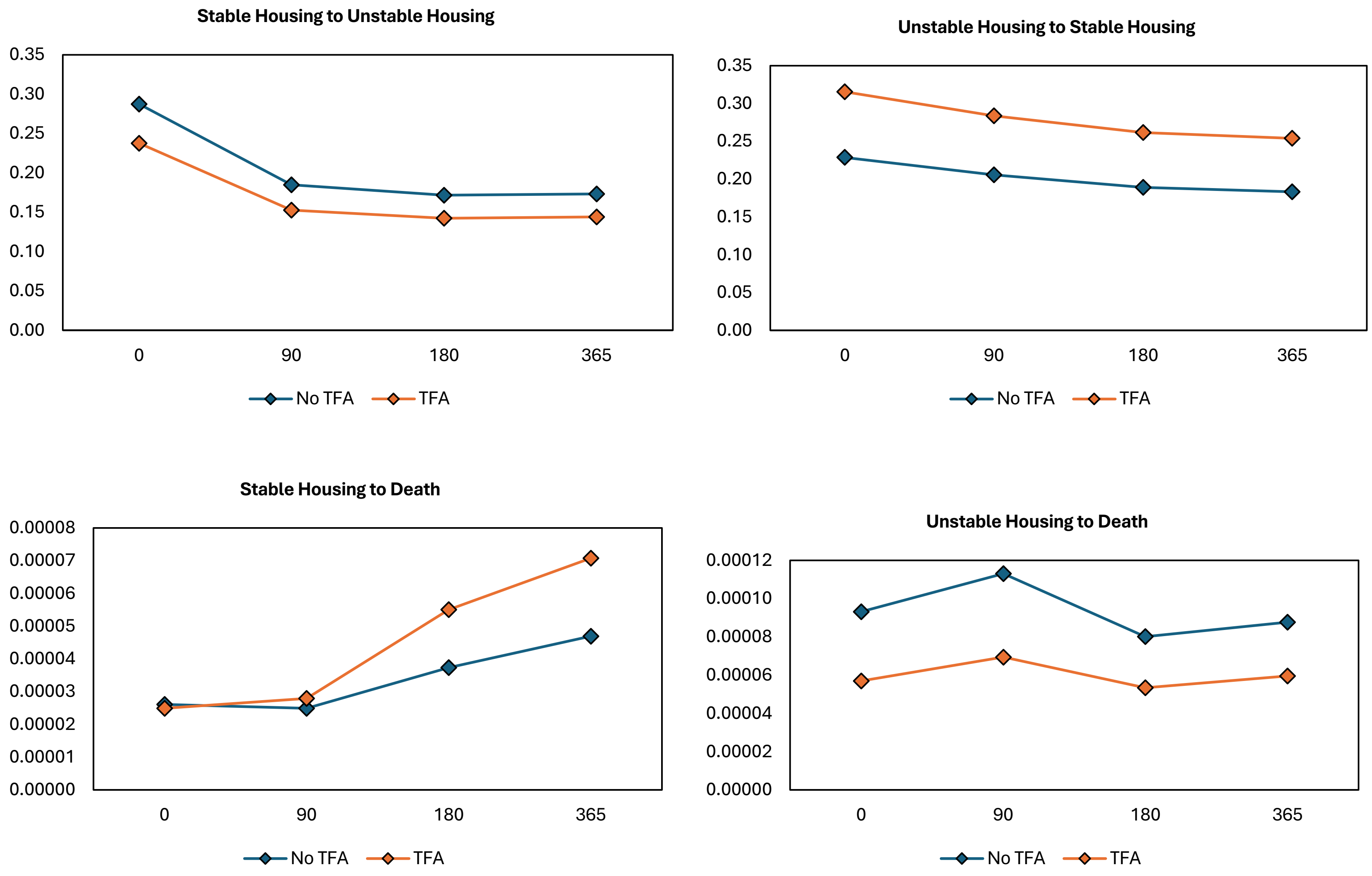

© 2024 Nelson RE et al. *JAMA Network Open.*

eAppendix. Details of Modeling Approach

The transition probabilities used in the simulation were estimating from electronic health record (EHR) data using a multi-state model (MSM). We used the *msm* package in R to perform our analysis.[1] Extensive details regarding the package and modeling methods are given in the package documentation. We provide a brief summary here and describe how we implemented a multi-state model for our analysis.

A Multi-State Model for Housing Status

Denote the *i-th* patient's state on the *t*- day *t* as $Y_i(t)$, $t=0, 1, 2, \ldots, \tau$, where τ=730 is the final day of follow-up. $Y_i(t)$ takes on values *1* for "stably housed", *2* for "unstably housed", and *3* for "Dead". Through the follow-up period, patients can remain in the same state, transition between homelessness and housing, or die. This three-state model is represented in eFigure 3.

© 2024 Nelson RE et al. *JAMA Network Open.*

eFigure 3. Multi-State Model Between Stable Housing, Unstable Housing, and Death

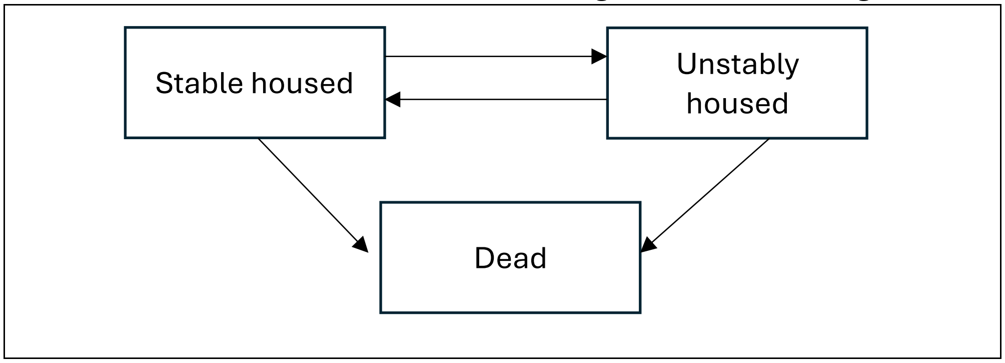

The process of moving through states is described through transition probabilities:

$$p_{jk}(s,t) = P(Y_i(t) = k | Y_i(s) = j)$$

Transition probabilities are assumed to have the Markov property, such that the transition probability at time *t* depends only on the previous state:

$$P(Y_i(t) = y | Y_i(t-1) = y_{t-1}, Y_i(t-2) = y_{t-2} \ldots Y_i(0) = y_0)$$
$$= P(Y_i(t) = y | Y_i(t-1) = y_{t-1})$$

Between two time points, the three-state model used in our analysis and shown in Figure S1 can be represented using a *transition probability matrix*, where the entry in the *j-th* row and *k-th* column corresponds to the probability of transitioning from state *j* to state *i*:

$$\boldsymbol{P}(s,t) = \begin{bmatrix} p_{11}(s,t) & p_{12}(s,t) & p_{13}(s,t) \\ p_{21}(s,t) & p_{22}(s,t) & p_{23}(s,t) \\ 0 & 0 & 1 \end{bmatrix}$$

The objective of our analysis was to estimate the 6 transition probabilities p_{jk}, $j \neq 3, k=1,2,3$, with the probability of transitioning from Death to stable housing (p_{31}) or unstable housing (p_{32}) defined to be 0.

Patients were not observed each day of the follow-up period; instead, most follow-up times were collected at irregular intervals when the patient presented to the VA for care. The assessment process is described in detail later. To account for the long gaps between measurements, we used a continuous-time modeling framework. In continuous-time MSM models, transitions are assumed to occur at any time point in a range of time, which may or may not be exactly observed in the data. The state transition process is modeled using *transition intensities*, which represent the instantaneous risk of transitioning from one state to another:

$$q_{jk}(t) = \lim_{\delta t \to 0} P(Y_i(t + \delta t) = k | Y_i(t) = j)/\delta t$$

The 3x3 matrix *Q(t)* contains the transition intensities at time *t*:

© 2024 Nelson RE et al. *JAMA Network Open.*

$$\boldsymbol{Q}(t) = \begin{bmatrix} -(q_{12}(t) + q_{13}(t)) & q_{12}(t) & q_{13}(t) \\ q_{21}(s,t) & -(q_{21}(t) + q_{23}(t)) & q_{23}(t) \\ 0 & 0 & 0 \end{bmatrix}$$

Where the diagonal terms are constrained to sum to 0 with the off diagonals. Transition intensities are estimated from data using maximum likelihood methods.

Transition intensities may change over time. For example, in our study, patients who enroll in SSVF may see an initial increase in the probability of transitioning into (or remaining in) stable housing, which could attenuate over time as patients face an increased risk of relapsing into homelessness or alternatively find long-term stable housing. A *time-inhomogeneous* defines intervals where the transition intensity may change at pre-specified points but remains constant in between. A time inhomgeneous model involves calculating the transition matrices over all of the time periods defined by the intervals.

Given two times *s* and *t, s<t*, and the transition corresponding to the time intervals between them, transition probabilities ***P****(s, t)* can be calculated using matrix operations. 95% confidence intervals for the intensities and probabilities can then be obtained from the observed information matrix. The *msm* package calculates transition probabilities and confidence intervals using the *pmatrix.msm* function; additional details are described in the *msm* documentation.

Estimating Transition Probabilities from Electronic Health Record Data
Our ultimate objective was to evaluate whether temporary financial assistance (TFA) was cost-effective by reducing homelessness and/or mortality in the two years after SSVF enrollment. To do this, we fit a model using administrative and EHR data to estimate the transition probabilities for the 3-state framework described above dependent on a patient's receipt of TFA.

Extracting patient states
Patient treatment information and housing states were extracted from administrative data and the VA EHR. First, treatment information including receipt of TFA and whether the patient was receiving rapid rehousing or homelessness prevention services were obtained from the Homelessness Management Information Systems (HMIS).

Next, we constructed a longitudinal dataset of patients' housing states over the two-year follow-up period where each row corresponded to a measurement of their housing status or their date of death. On day 0 (i.e., the date they enrolled in SSVF), the patients were assigned a status of "Housed" ($Y_i(0)=1$) if they were identified as receiving homelessness prevention services or a status of "Homeless" ($Y_i(0)=2$) if they were receiving rapid rehousing services. Death dates during the two years following SSVF entry were obtained from the VA Vital Status File. When a patient died before the end of the study, their follow-

© 2024 Nelson RE et al. *JAMA Network Open.*

up ended at time D_i, where D_i is the number of days between the patient's entry into SSVF and death. In this case, the patient's final entry in the dataset was $Y_i(D_i) = 3$.

In between SSVF entry and the end of a patient's follow-up, housing status was extracted from clinical notes using a natural language processing (NLP) system called ReHouSED.[2] The input for ReHouSED is clinical notes that have housing-related keywords related to housing. For each note, ReHouSED identifies phrases related to sheltered or unsheltered homelessness (e.g., "sleeps in the park" or "has a bed in the shelter"); housing-related needs or risk of homelessness ("needs help with housing" or "can't pay rent"); or stable housing ("signed a lease" or "doing well in her apartment"). Each concept is assigned linguistic attributes such as negation ("*not* homeless") or temporality ("*previously had an* apartment"). Each note processed by ReHouSED is assigned a document classification of "Stable housing", "Unstable housing", or "Unknown". A note is classified as "Stable housing" if there was at least one mention of current independent housing. If there is no mention of stable housing but there is a mention of either homelessness or ongoing housing issues, the note is classified as "Unstable housing". Otherwise, the note is determined to not be informative regarding current housing status and is classified as "Unknown" and were excluded from the dataset. ReHouSED was developed and validated using notes from SSVF enrollees[2]. In the corpus of data used to evaluate ReHouSED, positive predictive value was estimated to be 64.0% and 78.3% for "Stable" and "Unstable", respectively; sensitivity was estimated to be 62.7% and 64.9%. We discuss the potential impact of measurement error on our analysis below.

For the patients in our cohort, we extracted all notes containing housing keywords in the two years after SSVF entry. Patients were assigned to housing-related states on days when the patient had at least one note processed by ReHouSED and assigned a classification other than "Unknown". If at least half of these notes on a day were "Unstable", the patient was assigned a state of $Y_i(t)=2$; otherwise, if the majority of notes were classified as "Stably housed", the patient was assigned a state of $Y_i(t)=1$. On days that the patient did not have a visit or did not have a note classified by ReHouSED as "Unstably" or "Stably housed", patients did not have a measurement of housing status.

Model specification

We fit two multistate models using our longitudinal dataset. In the first model, we assumed that the probability of a patient transitioning between states at two time points depended on their state at the first time point, whether they had received TFA, and the number of days since a patient's entry into SSVF. To evaluate the effect of TFA on transition probabilities, A_i, indicating receipt of TFA as an independent model in the state transition model. Additionally, as discussed above, we hypothesized that transition probabilities may change over the study period and fit a time inhomogeneous model with days *90*, *180*, and *365* for change points in the intensities. Previous studies have shown that the average length of enrollment in SSVF is around 90 days;[3] thus, the first interval from Day *0* to Day *90* represent the time when a patient was expected to be most actively involved in SSVF and

© 2024 Nelson RE et al. *JAMA Network Open.*

receiving assistance. Days *90-180* represent an immediate post-treatment period, when patients who exited to stable housing could either relapse into homelessness or maintain stable housing, and patients who remained enrolled in SSVF or exited to unstable housing may continue being homeless or eventually exit. The final two time intervals represent longer-term periods of housing status changes.

After estimating transition intensities across these four intervals, we obtained the probability of transitioning between states on adjacent days in each time period. That is, we calculated:

$$p_{jk}^{(a)}(t, t+1) = P(Y_i(t+1) = k | Y_i(t) = j | A_i = a)$$

for *a=0, 1*; *j=1,2*; *k=1,2,3*; and *t=0, 90, 180, 365*,

Our second model adjusted for whether a patient had enrolled in SSVF to receive rapid rehousing or homelessness prevention services. That is, letting R_i=1 if a patient was enrolled in rapid rehousing services, we calculated:

$$p_{jk}^{(a,r)}(t, t+1) = P(Y_i(t+1) = k | Y_i(t) = j | A_i = a, R_i = r)$$

for *r=0,1* and *a, j, k,* and *t* the same as Model #1.

Model validation

To validate our model, we compared the predicted probabilities of each state over the two-year follow-up period that were obtained using the estimated transition probabilities from our TFA vs No TFA model with estimates from a marginal longitudinal model used in other studies[4,5]. Marginal models do not specify the covariance structure between different time points; in contrast, the multistate Markov model relies on the assumption that a patient's next state depends on past history only through their current state, a stronger assumption that could lead to biased estimates if it is violated in the data. Additionally, the marginal models we used is less computationally expensive and restrictive than a multistate Model, allowing us to more flexibly model housing status as a function of time and TFA.

The marginal model for the overall TFA vs no TFA analysis was specified as follows. Define $\mu_{ij}(t) = P(Y_i(t) = j | A_i)$ to be the probability that patient *i* be in state *j* on day *t*, *j=1,2,3* given their TFA status. Next, let D_i be the patient's death time. If the patient survives to the end of the study period, then the exact death time is not known, but it is known that $D_i > \tau$. The probability a patient is in State 3 on day *t* can be written as $\mu_{i3}(t) = P(Y_i(t) = 3 | A_i) = P(D_i \leq t | A_i)$.

Next, define $\pi_{ij}(t) = P(Y_i(t) = j | A_i, D_i > t), j = 1,2$ to be the conditional probability of being either stably housed (*Y_i=1)* or unstably housed (*Y_i=2*) on day *t* given that the patient is alive on day *t*. Then we can write $\mu_{i2}(t)$ as:

$$\mu_{i2}(t) = P(Y_i(t) = j | A_i) = P(Y_i(t) = j | D_i > t, A_i) P(D_i > t)$$
$$= \pi_{i2}(t)\big(1 - \mu_{i3}(t)\big)$$

© 2024 Nelson RE et al. *JAMA Network Open.*

We estimated the death probabilities $\mu_{i3}(t)$ non-parametrically as the proportion of patients in the same treatment group as patient *i* who had died on or before day *t*:

$$\hat{\mu}_{i3(t)} = \hat{P}(Y_i(t) = 3|A_i = a) = \frac{1}{\sum_{l=1}^{n} I(A_l = a)} I(A_i = a, D_i \le t)$$

The conditional probability of being unstably housed $\pi_{12}(t)$ was estimated using a logistic regression model. The dependent variable was whether the patient was observed to be unstably housed on day *t*. The independent variables were treatment status, time since entry with cubic splines, and an interaction term. Cubic splines, denoted as *S(t)*, were fit with knots on days 90, 180, and 365, similar to the multistate model.

$$\text{logit}\pi_{i2}(t) = \beta_0 + \beta_1 A_i + \beta_2 S(t) + \beta_3\big(A_i \times S(t)\big)$$

After fitting these models, we estimated the probability of being alive and homeless each day for TFA and non-TFA recipients:

$$\hat{\mu}_{i2}(t) = \hat{\pi}_{i2}(t)\big(1 - \hat{\mu}_{i3}(t)\big)$$

The marginal probability of being alive *and* stably housed on day *t* was estimated as:

$$\hat{\mu}_{i1}(t) = 1 - \hat{\mu}_{i2}(t) - \hat{\mu}_{i3}(t)$$

An assumption of the multi-state model implemented in *msm* is that the assessment times at which a patient's state is measured is uninformative. This would be violated, for example, if patients who were homeless visited the VA and had housing-related notes more frequently. Past studies have addressed this in analyses of housing outcomes for SSVF enrollees; [4,5] however, in the context of a multistate model this remains challenging and is not addressed in the *msm* package. To assess whether informative assessment times were an issue in our dataset and to what extent it might affect the analysis, we repeated the analysis above with the homelessness model weighted using inverse intensity weighting (IIW), which uses patient factors including baseline characteristics and past homelessness history to adjust for irregular and possibly informative visit times.[6] This approach is described in detail elsewhere.[4,5] Using the inverse intensity-weighted models, we obtained adjusted probabilities of homelessness and housing:

$$\hat{\mu}_{i2}^{(IIW)}(t) = \hat{\pi}_{i2}^{(IIW)}(t)\big(1 - \hat{\mu}_{i3(t)}\big)$$

$$\hat{\mu}_{i1}^{(IIW)}(t) = 1 - \hat{\mu}_{i2}^{(IIW)}(t) - \hat{\mu}_{i3}(t)$$

95% confidence bands for each of the curves were calculated using bootstrapping.

eFigure 4 shows the results of the Markov model used for the cost-effectiveness analyses along with the unweighted marginal longitudinal model (Panel A) and the weighted marginal longitudinal model (Panel B).

© 2024 Nelson RE et al. *JAMA Network Open.*

eFigure 4. Comparison of State Probabilities from Markov Model With Weighted and Unweighted Marginal Longitudinal Models

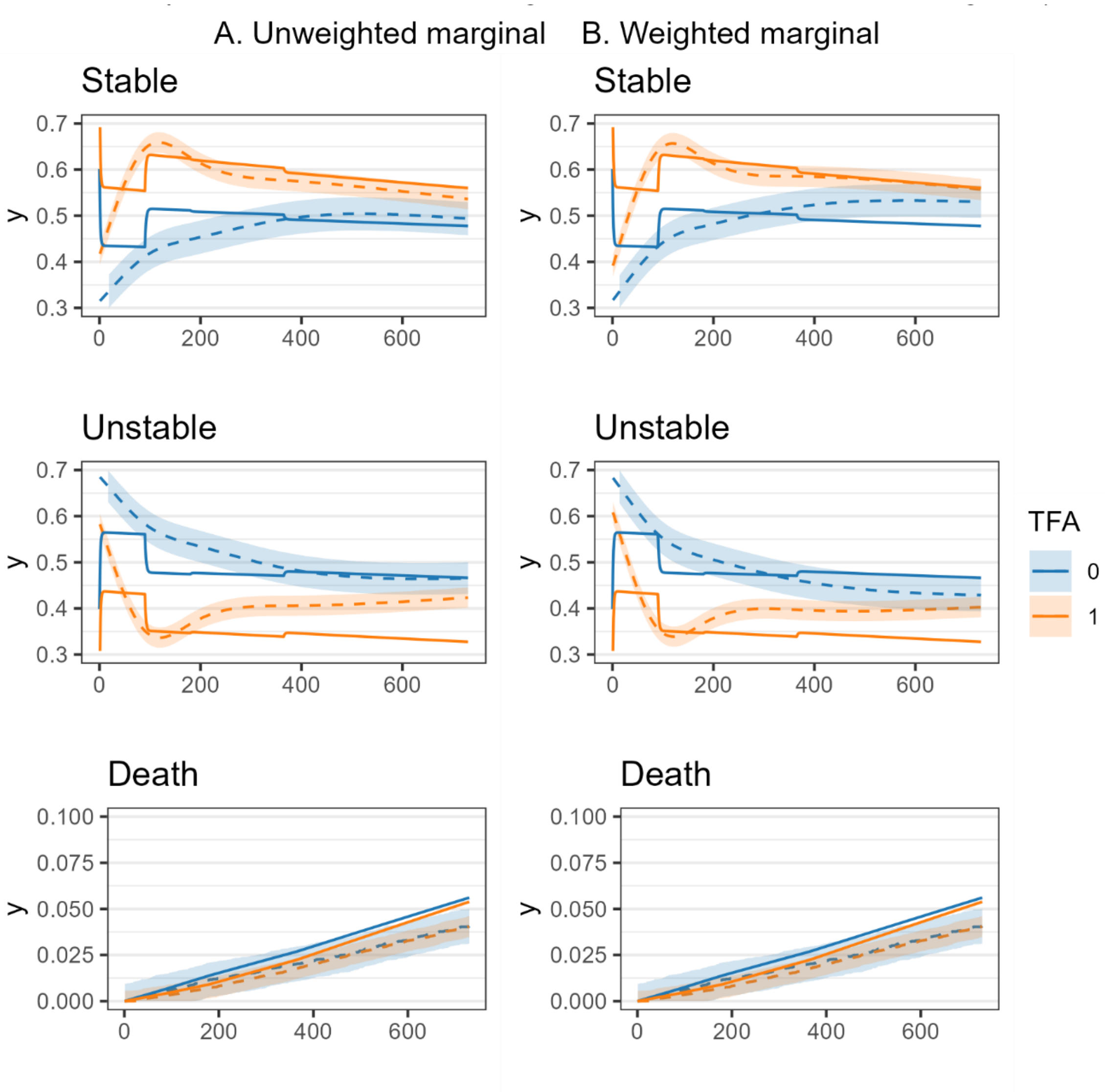

Note: solid lines indicate Markov model output, dashed lines indicate marginal model output

© 2024 Nelson RE et al. *JAMA Network Open.*

Limitations

There are several limitations to our modeling strategy. First, the definition of homelessness implemented by ReHouSED is coarse and includes several forms of housing instability including sheltered homelessness, unsheltered homelessness, and general housing issues. Future work should consider more precise definitions of homelessness, as well as definitions that differentiate between sheltered and unsheltered homelessness. Second, our multistate model ignores the potentially informative assessment times of measurements of housing status. As shown by the marginal model, this may cause a slight bias upward in the estimates of housing instability. Third, we do not address measurement error in our model and observed transitions between states do not perfectly correspond to true transitions between housing states. Additionally, measurement error in multistate models can lead to violations of the Markov assumption in the transition intensities as the previous state is not adequately controlled for.[7] One potential solution that should be addressed in future work is methods such as Hidden Markov Models that incorporate uncertainty due to measurement error and aim to learn the transition probabilities of the true, unobserved states. However, this was beyond the scope of our work.

© 2024 Nelson RE et al. *JAMA Network Open.*

eReferences.

1. Jackson C. Multi-state modelling with R: the msm package. Cambridge, UK [Internet]. 2007;1–53. Available from: ftp://218.193.60.3/software/rsoft/msm/doc/msm-manual.pdf
2. Chapman AB, Jones A, Kelley AT, Jones B, Gawron L, Montgomery AE, et al. ReHouSED: A novel measurement of Veteran housing stability using natural language processing. J Biomed Inform [Internet]. 2021;122:103903. Available from: https://doi.org/10.1016/j.jbi.2021.103903
3. Nelson RE, Byrne TH, Suo Y, Cook J, Pettey W, Gundlapalli A V., et al. Association of Temporary Financial Assistance With Housing Stability Among US Veterans in the Supportive Services for Veteran Families Program. JAMA Netw Open. 2021 Feb 10;4(2):e2037047.
4. Chapman AB, Scharfstein D, Byrne TH, Montgomery AE, Suo Y, Effiong A, et al. Temporary Financial Assistance Reduced The Probability Of Unstable Housing Among Veterans For More Than 1 Year. Health Aff [Internet]. 2024 Feb 1;43(2):250–9. Available from: https://www.healthaffairs.org/doi/abs/10.1377/hlthaff.2023.00730
5. Chapman AB, Scharfstein DO, Montgomery AE, Suo Y, Effiong A, Velasquez T, et al. Using natural language processing to study homelessness longitudinally with electronic health record data subject to irregular observations. In: AMIA Annu.al Symposium Proceedings 2023 (in press). 2023.
6. Lin H, Scharfstein DO, Rosenheck RA. Analysis of longitudinal data with irregular, outcome-dependent follow-up. J R Stat Soc Series B Stat Methodol [Internet]. 2004;66(3):791–813. Available from: https://www.jstor.org/stable/3647506
7. Yi G. Statistical Analysis with Measurement Error or Misclassication [Internet]. New York, New York, USA: Springer; 2017. Available from: https://link.springer.com/book/10.1007/978-1-4939-6640-0

© 2024 Nelson RE et al. *JAMA Network Open.*
