# Supplementary material for: Cost-Effectiveness of Temporary Financial Assistance for Veterans Experiencing Housing Instability
Source: JAMA Netw Open. 2024 Nov 5;7(11):e2443396. doi: 10.1001/jamanetworkopen.2024.43396 (PMC11539017; doi:10.1001/jamanetworkopen.2024.43396)
Supplement: Supplement 2. — Data Sharing Statement [file jamanetwopen-e2443396-s002.pdf]

Data Sharing Statement

Nelson. Cost-Effectiveness of Temporary Financial Assistance for Veterans Experiencing Housing Instability. *JAMA Netw Open*. Published November 05, 2024. doi:10.1001/jamanetworkopen.2024.43396

Data
Data available: Yes
Data types: Other (please specify)
Additional Information: Input parameters for our computer simulation model
How to access data: The input parameters for our decision analysis are contained in Table 1 and Supplemental Figures 1 and 2 of the submitted manuscript
When available: With publication

Supporting Documents
Document types: None

Additional Information
Who can access the data: Anyone requesting the data
Types of analyses: For any purpose
Mechanisms of data availability: Without investigator support
